# Gestational diabetes and its influence on early neurodevelopment in infants: a retrospective analysis

**DOI:** 10.3389/fped.2026.1756844

**Published:** 2026-08-19

**Authors:** Dan Liu, Xiaoxia Chen, Yili Zhang, Biao Chen

**Affiliations:** Department of Child Health, Shaoxing Maternity and Child Health Care Hospital, Shaoxing, Zhejiang, China

**Keywords:** Gestational diabetes mellitus, cognitive development, infancy, BSID-CR, developmental quotient, neurodevelopmental outcomes

## Abstract

**Background & objective:**

Gestational diabetes mellitus (GDM) is associated with adverse maternal and neonatal outcomes, but its impact on early cognitive function and neurodevelopment remains uncertain. This study evaluates cognitive and developmental outcomes in infants born to mothers with GDM compared to non-diseased mothers.

**Methods:**

A retrospective cohort study was conducted in infants born to mothers with or without GDM. Cognitive development was assessed using the Bayley Scales of Infant Development for Chinese Revision (BSID-CR), and developmental quotient (DQ) scores were extracted for analysis. Developmental concerns were identified from infant assessment records. Logistic and linear regression analyses were applied to determine associations between GDM and early developmental outcomes, adjusting for maternal and neonatal variables.

**Results:**

A total of 292 infants (145 born to mothers with GDM vs. 147 controls) were eligible for study. Maternal and neonatal characteristics were comparable between the GDM and control groups. No significant differences were observed between the groups in developmental concern occurrence (32.0% vs. 33.1%, *p* = 0.837) or corrected BSID-CR DQ score [median 94 (IQR, 83–103) vs. 96 (IQR, 85–102), *p* = 0.813]. The revised logistic model showed no independent association between GDM and developmental concern occurrence (OR = 1.090, 95% CI 0.602–1.972, *p* = 0.776), and the revised linear model showed no independent association between GDM and corrected BSID-CR DQ score (*B* = −0.069, 95% CI −3.807 to 3.670, *p* = 0.971).

**Conclusion:**

Infants born to mothers with GDM demonstrated comparable cognitive and developmental outcomes to controls. Moreover, GDM may not independently impact cognitive development in infancy when managed appropriately.

## Introduction

Gestational diabetes mellitus (GDM) is defined as glucose intolerance first recognized during pregnancy. It is considered as one of the most common pregnancy-related complications (1). Its global prevalence is increasing over past decades, with an estimated 1 in 6 live births exposed to maternal hyperglycemia and about 42% of all pregnant women are expected to develop GDM by 2030 (2–4). This increased prevalence of GDM is attributed to factors such as, increasing maternal age, obesity, and different lifestyles (5, 6). GDM is associated with risks for both mother and infant including increased likelihood of complications such as preeclampsia, shoulder dystocia, excessive fetal growth, premature birth, and cesarean delivery (6, 7).

Recently, some reports found higher risks of neonatal complications including hypoglycemia and respiratory distress in newborns of GDM mothers (6, 8). Additionally, GDM also increases the long-term risk of type 2 diabetes and cardiovascular disease in mothers, as well as obesity and metabolic disorders (7, 9). These medical complications and risks indicate that GDM is a significant medical concern. The impact of GDM on neurodevelopment of infants, particularly cognitive development in early life is still unclear. The current evidence suggests that maternal hyperglycemia could negatively impact the developing fetal brain (6). Several studies have reported associations between GDM exposure and adverse neurodevelopmental outcomes in early childhood including poor cognitive function, language difficulties, and higher rates of neurodevelopmental abnormalities such as autism spectrum disorder (ASD) and attention-deficit/hyperactivity disorder (ADHD) (10–12).

There are possible mechanisms in which GDM may influence neurodevelopment in infants since maternal hyperglycemia leads to fetal hyperinsulinemia and an abnormal intrauterine metabolic environment (13). Fluctuations in blood glucose levels, placental dysfunction related to GDM and exposure to excess growth factors can result in fetal tissue hypoxemia and nutrient deficiencies, which negatively influence myelination and in the developing brain and nervous system (14, 15). Additionally, infants of diabetic pregnancies experience higher oxidative stress and pro-inflammatory cytokine exposure *in utero*, raising the risk of neuropsychiatric disorders (16).

Monitoring the process of development in infancy and early childhood and understanding the underlying mechanisms are crucial as infancy represents a critical phase for brain development, characterized by rapid neuronal growth that affects the later cognitive and behavioral function (17, 18). Abnormal intrauterine conditions during this period can have persistent consequences at long-term including learning difficulties or neurodevelopmental disorders. Conversely, the high plasticity of brain in infancy offers an opportunity for early intervention in case of early recognition of risks (17, 19).

Recently, research field focuses on earliest manifestations of any neurodevelopmental impact of GDM, facilitating timely support or therapeutic strategies to improve long-term trajectories (20, 21). These insights highlight the great clinical importance of first years of life for guiding pediatric surveillance and early childhood interventions. Therefore, this comparative study evaluates cognitive and neurodevelopmental outcomes in infants born to mothers with GDM compared to non-diseased mothers.

## Methods

### Study design

This was a retrospective cohort study involving all data of infants born to mothers with GDM at Shaoxing Maternity and Child Health Care Hospital between January 1st, 2022- December 31st, 2023 to evaluate the impact of GDM on neurodevelopmental outcomes in addition to association of maternal and gestational variables with cognitive function. All maternal and infant data included in this retrospective cohort study were obtained exclusively from Shaoxing Maternity and Child Health Care Hospital electronic medical records.

The study gained approval from the institutional review board (IRB) (Ethical approval number:2025-060-01), and all patient data were anonymized to protect patient confidentiality.

### Participants

Infants were included in this study based on the following criteria: Infants aged between 6 and 24 months, born to mothers who had a confirmed diagnosis of GDM. Additionally, infants should have available complete medical records and neurodevelopmental assessment results. Mothers of eligible infants were required to receive prenatal care during the pregnancy follow up with no significant chronic conditions or complications. We excluded infants with genetic or congenital disorder like down syndromes that could negatively impact cognitive function and development. Moreover, infants with incomplete medical documentation or cognitive assessment data were also excluded from analysis. Infants born to mothers with pregestational diabetes including Type 1 and type 2 diabetes were excluded. Furthermore, infant born to mothers without diabetes mellitus fulfilling the eligibility criteria served as control group.

### Data collection and clinical characteristics

We retrospectively collected maternal demographic data, including maternal age, body mass index (BMI), medical history, and history of psychiatric disorders, from medical records. Obstetric and neonatal variables, including parity, mode of delivery, gestational age, breastfeeding duration, perinatal complications, respiratory distress, and neonatal convulsions, were also extracted from the medical records.

In addition to demographic and obstetric variables, detailed gestational diabetes–related data were extracted from medical records. These included oral glucose tolerance test (OGTT) values at diagnosis, HbA1c levels during pregnancy, classification of GDM severity based on glucose thresholds, and treatment modality (diet-controlled vs. pharmacologic therapy including insulin). Glycemic control status during pregnancy was categorized according to documented clinical targets. These variables were included in subgroup and multivariable analyses to evaluate whether severity or metabolic control modified neurodevelopmental outcomes.

### Neurodevelopmental outcomes

Neurodevelopmental and cognitive outcomes were evaluated using the Bayley Scales of Infant Development for Chinese Revision (BSID-CR) and collected from infant assessment records. Developmental quotient (DQ) scores were interpreted according to the standardized classification used in the hospital registry: normal developmental range was defined as DQ 85–115, above-average development as DQ >115, borderline developmental status as DQ 70–84, and developmental delay as DQ <70. Extremely low development, defined as DQ <50, was considered a subgroup within the developmental delay category.

### Statistical analysis

Continuous variables were described using medians and interquartile ranges (IQR), while categorical variables were reported as frequencies and percentages. Normality was assessed with the Shapiro-Wilk test. Group comparisons for continuous variables were conducted using the Mann-Whitney U test, and categorical variables were compared using Pearson's chi-square test or Fisher's exact test when expected cell counts were low. Multivariable logistic regression was used to assess developmental concern occurrence, and multivariable linear regression was used to assess corrected BSID-CR DQ score. Variables not included in the finalized reporting plan were omitted from the final descriptive presentation and adjusted models.

## Results

### Maternal demographic and clinical characteristics

The maternal demographic and clinical characteristics were comparable between the control group (*n* = 147) and the GDM group (*n* = 145). No statistically significant differences were observed in maternal age (median 28 years in both groups, *p* = 0.98), maternal BMI (median 26 and 26.1 kg/m2, respectively, *p* = 0.904), or racial distribution, parity, breastfeeding duration, pesticide exposure, antibiotic intake, or psychiatric history (Table 1).

**Table 1:** Maternal demographic and clinical characteristics among control and gestational diabetes mellitus groups.

Variable	Category	Control group	Gestational DM group	*p*-value	Test
Maternal age at pregnancy, median (Q1, Q3)		28 (23, 34)	28 (23, 34)	0.981	Mann–Whitney U test
Maternal BMI, median (Q1, Q3)		26 (23.2, 28.9)	26.1 (23.1, 29)	0.904	Mann–Whitney U test
Parity, median (Q1, Q3)		1 (0, 2)	1 (0, 2)	0.153	Mann–Whitney U test
Breastfeeding duration (months), median (Q1, Q3)		4 (1, 9)	5 (2, 10)	0.256	Mann–Whitney U test
Pesticide exposure, *n* (%)	No	116 (85.9)	117 (85.4)	0.902	Pearson chi-square test
	Yes	19 (14.1)	20 (14.6)		
Antibiotic intake, *n* (%)	No	101 (69.7)	101 (70.1)	0.929	Pearson chi-square test
	Yes	44 (30.3)	43 (29.9)		
Maternal psychiatric history, *n* (%)	No	131 (89.1)	128 (88.3)	0.821	Pearson chi-square test
	Yes	16 (10.9)	17 (11.7)		

Analysis set: total *n* = 292, control *n* = 147, GDM *n* = 145.

Lifestyle-related variables not included in the finalized reporting plan were omitted from the descriptive presentation and adjusted models.

### Neonatal characteristics

Neonatal characteristics, including gestational age (median 38 months for both groups, *p* = 0.791), birth weight (median 3,226 g control group, 3,200 g GDM group, *p* = 0.608), mode of delivery, gender distribution, respiratory distress, and neonatal convulsions, did not significantly differ between the two groups (Table 2).

**Table 2:** Neonatal characteristics by maternal diabetes status.

Variable		Control group	Gestational DM group	*p*-value
Gestational age (months), median (Q1, Q3)		38 (35, 40)	38 (35, 40)	0.791^a^
Mode of delivery, *n* (%)	Vaginal	76 (54.7)	71 (50.7)	0.508^b^
	Cesarean	63 (45.3)	69 (49.3)	
Child Gender, *n* (%)	Female	75 (51)	71 (49)	0.725^b^
	Male	72 (49)	74 (51)	
Birth weight (grams), median (Q1, Q3)		3,226 (2,855, 3,514)	3,200 (2,981, 3,386)	0.608^a^
Respiratory distress, *n* (%)	No	124 (84.4)	125 (86.2)	0.655^b^
	Yes	23 (15.6)	20 (13.8)	
Neonatal convulsions, *n* (%)	No	132 (89.8)	130 (89.7)	0.968^b^
	Yes	15 (10.2)	15 (10.3)	

a *p* value was calculated by Mann–Whitney *U* test.

b *p* value was calculated by Pearson's *χ*^2^ test.

### Neurodevelopmental outcomes

**Figure 1:**
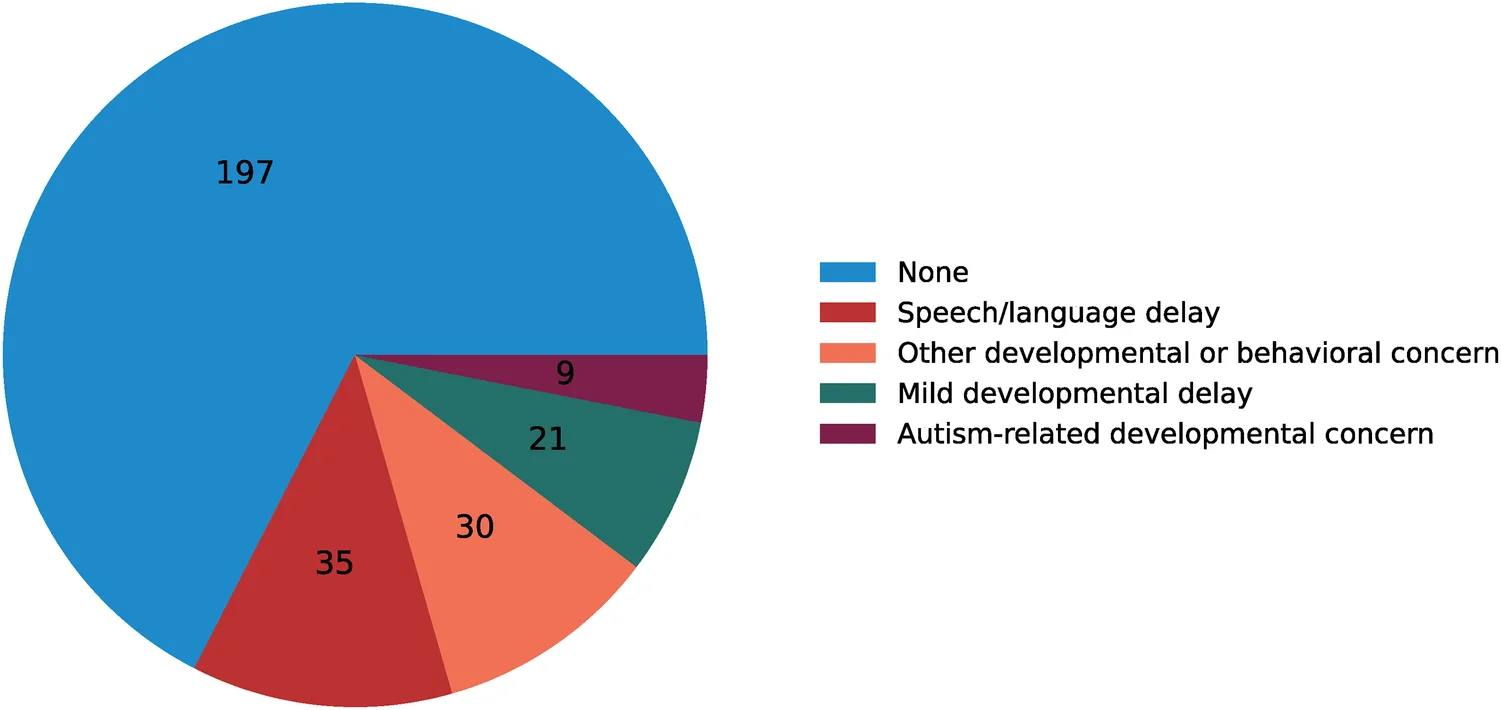
Distribution of developmental concerns in the total study population.

**Figure 2:**
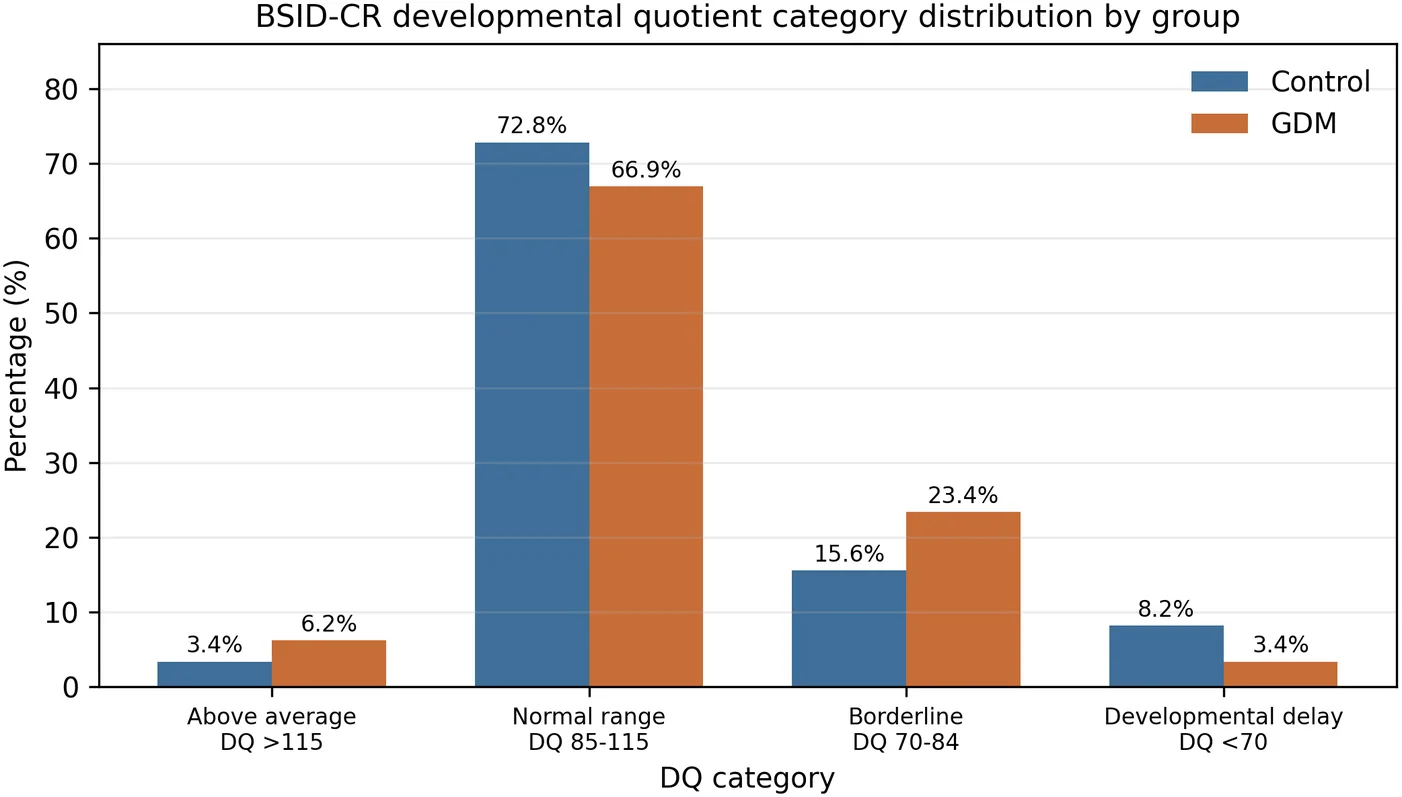
Distribution of BSID-CR developmental quotient (DQ) categories by maternal GDM status. Extremely low development (DQ <50) was included within the developmental delay category and was not plotted as a separate mutually exclusive category.

The overall distribution of developmental concerns within the entire study population indicated that the most prevalent concerns were speech/language delay, other developmental or behavioral concern, mild cognitive delay, and autism-related developmental concern (Figure 1). According to the corrected BSID-CR DQ classification, most infants were within the normal developmental range, while smaller proportions were classified as borderline developmental status or developmental delay. Extremely low development (DQ <50) was included within the developmental delay category and was not counted as a separate mutually exclusive category (Figure 2).

Overall, there was no significant difference in the occurrence of developmental concerns between the control and GDM groups (32.0% vs. 33.1%, respectively, *p* = 0.837). The median corrected BSID-CR DQ score was 96 (85, 102) in the control group and 94 (83, 103) in the GDM group, with no statistically significant between-group difference (*p* = 0.813). The distribution of the four mutually exclusive DQ categories also did not differ significantly between groups (*p* = 0.085) (Table 3).

**Table 3:** Developmental outcomes and corrected BSID-CR developmental quotient categories.

Variable	Category	Control group	Gestational DM group	*p*-value	Test
Child developmental concern, *n* (%)	None	100 (68.0)	97 (66.9)	0.837	Pearson chi-square test; any developmental concern vs. none
Child developmental concern, *n* (%)	Autism-related developmental concern	7 (4.8)	2 (1.4)		
Child developmental concern, *n* (%)	Mild developmental delay	9 (6.1)	12 (8.3)		
Child developmental concern, *n* (%)	Speech/language delay	14 (9.5)	21 (14.5)		
Child developmental concern, *n* (%)	Other developmental or behavioral concern	17 (11.6)	13 (9.0)		
BSID-CR DQ score, median (Q1, Q3)		96 (85, 102)	94 (83, 103)	0.813	Mann–Whitney U test
DQ category, *n* (%)	Above-average development, DQ >115	5 (3.4)	9 (6.2)	0.085	Pearson chi-square/Fisher exact test for four mutually exclusive DQ categories
	Normal developmental range, DQ 85–115	107 (72.8)	97 (66.9)		
	Borderline developmental status, DQ 70–84	23 (15.6)	34 (23.4)		
	Developmental delay, DQ <70	12 (8.2)	5 (3.4)		
	Including extremely low development, DQ <50	2 (1.4)	3 (2.1)		Descriptive subgroup row; included within developmental delay, DQ <70

Extremely low development (DQ <50) was considered a subgroup of developmental delay (DQ <70) and was not counted as a separate mutually exclusive category.

Analysis set: total *n* = 292, control *n* = 147, GDM *n* = 145.

### Gestational diabetes severity and treatment characteristics

Among mothers diagnosed with GDM (*n* = 145), the majority were managed with dietary modification alone, while a smaller proportion required pharmacologic therapy. Median HbA1c levels during pregnancy were within clinically controlled ranges. OGTT values at diagnosis demonstrated predominantly mild-to-moderate hyperglycemia according to standard diagnostic thresholds.

Subgroup analyses comparing diet-controlled vs. pharmacologically treated GDM showed no statistically significant differences in offspring cognitive disorder prevalence or Bayley-4 scores. Similarly, stratification according to glycemic control status did not demonstrate significant associations with neurodevelopmental outcomes (Supplementary Tables S1, S2).

### Multivariate binary regression analysis of developmental concern occurrence

In the revised multivariable logistic regression model, GDM was not independently associated with developmental concern occurrence (OR = 1.090, 95% CI 0.602–1.972, *p* = 0.776). Other analyzed variables, including maternal age, BMI, parity, breastfeeding duration, pesticide exposure, antibiotic intake, gestational age, delivery mode, psychiatric history, child sex, birth weight, respiratory distress, and neonatal convulsions, were not significantly associated with developmental concern occurrence (Table 4).

**Table 4:** Multivariable logistic regression analysis predicting cognitive disorder occurrence.

Variable	*B*	SE	*p*-value	OR	95% CI lower for OR	95% CI upper for OR
Maternal age at pregnancy	0.013	0.021	0.535	1.013	0.972	1.056
Maternal BMI	−0.068	0.036	0.057	0.934	0.87	1.002
Group (GDM vs. control)	0.086	0.302	0.776	1.09	0.602	1.972
Parity	0.056	0.116	0.632	1.057	0.842	1.327
Breastfeeding duration (months)	0.012	0.027	0.663	1.012	0.96	1.066
Pesticide exposure (yes)	0.589	0.425	0.166	1.802	0.784	4.144
Antibiotic intake (yes)	0.38	0.327	0.245	1.463	0.77	2.779
Gestational age (weeks)	−0.027	0.041	0.516	0.973	0.898	1.056
Mode of delivery (Cesarean)	0.023	0.314	0.941	1.024	0.553	1.893
Maternal psychiatric history (yes)	0.365	0.491	0.457	1.441	0.551	3.772
Child gender (male)	−0.425	0.31	0.171	0.654	0.356	1.202
Birth weight (grams)	0	0	0.269	1	0.999	1
Respiratory distress (yes)	−0.208	0.44	0.637	0.812	0.343	1.925
Neonatal convulsions (yes)	0.337	0.5	0.501	1.4	0.526	3.73
Constant	2.884	2.394	0.228	17.888	0.164	1,950.481

GDM remained non-significant: OR = 1.090, 95% CI 0.602–1.972, *p* = 0.776.

Lifestyle-related variables not included in the finalized reporting plan were omitted from the adjusted model.

### Multivariate linear regression analysis of corrected BSID-CR DQ score

In the revised multivariable linear regression model based on the corrected BSID-CR DQ score and the finalized covariate set, GDM was not independently associated with BSID-CR DQ score (*B* = −0.069, 95% CI −3.807 to 3.670, *p* = 0.971). No covariates in the revised model reached statistical significance (Table 5).

**Table 5:** Multivariable linear regression analysis of predictors of corrected BSID-CR DQ score.

Variable	*B*	Std. error	Standardized beta	*p*-value	95% CI lower for *B*	95% CI upper for *B*
Constant	80.545	15.009		<0.001	50.951	110.138
Maternal age at pregnancy	0.168	0.132	0.090	0.206	−0.093	0.428
Maternal BMI	0.065	0.222	0.021	0.77	−0.373	0.503
Group (GDM vs. control)	−0.069	1.896	−0.003	0.971	−3.807	3.67
Parity	−0.176	0.737	−0.017	0.811	−1.63	1.277
Breastfeeding duration (months)	−0.18	0.165	−0.078	0.276	−0.506	0.145
Pesticide exposure (yes)	−2.946	2.756	−0.075	0.286	−8.381	2.489
Antibiotic intake (yes)	−2.807	2.09	−0.094	0.181	−6.927	1.314
Gestational age (weeks)	−0.032	0.262	−0.008	0.903	−0.549	0.486
Mode of delivery (Cesarean)	−2.658	1.973	−0.097	0.179	−6.548	1.231
Maternal psychiatric history (yes)	−1.926	3.167	−0.043	0.544	−8.171	4.319
Child gender (male)	0.37	1.946	0.014	0.849	−3.466	4.207
Birth weight (grams)	0.004	0.003	0.106	0.134	−0.001	0.009
Respiratory distress (yes)	−0.453	2.709	−0.012	0.867	−5.796	4.889
Neonatal convulsions (yes)	1.991	3.228	0.043	0.538	−4.373	8.356

GDM remained non-significant: *B* = −0.069, 95% CI −3.807 to 3.670, *p* = 0.971.

Lifestyle-related variables not included in the finalized reporting plan were omitted from the adjusted model.

## Discussion

This retrospective study evaluated the impact of GDM on offspring early developmental outcomes. The pooled analysis of infants revealed no statistically significant differences in the occurrence of developmental concerns between infants born to mothers with GDM and those born to mothers without GDM. Similarly, corrected BSID-CR DQ scores were comparable between groups. Multivariate analyses further confirmed that GDM was not independently associated with developmental concern occurrence or corrected BSID-CR DQ score.

These findings align with previously published studies that challenge the notion of a direct link between GDM and long-term neurodevelopmental deficits. For instance, Ornoy et al., observed that the association between maternal GDM and child cognitive ability weakened after adjusting for confounders such as maternal education and socioeconomic status (24). Similarly, Nomura et al., found that after controlling for perinatal complications and environmental variables, GDM had a minimal independent effect on cognitive outcomes since children exposed to maternal GDM alone did not exhibit a significantly elevated risk for developing ADHD while maternal GDM combined with low socioeconomic states showed greater risk (25). On the other hand, Faleschini et al., reported that children exposed to higher maternal blood glucose levels during pregnancy showed more externalizing behaviors such as aggression and hyperactivity at ages of 3–5 years old with no obvious link between maternal glucose levels and internalizing behaviors (26). The discrepancy of findings may be attributed to different age groups, study designs and assessment tools. Potential neurodevelopmental effects associated with GDM exposure may manifest beyond cognitive performance, including social-emotional regulation and behavioral outcomes, which may become more apparent at older ages.

Importantly, among 292 infants born to mothers with and without GDM, we did not observe significant differences in neonatal characteristics including birth weight, gestational age, or mode of delivery. These factors are considered mediators of developmental outcomes. Furthermore, our analysis showed no elevated risk for developmental concerns in the GDM group. These observations are consistent with results from prior studies indicating limited independent associations after adjustment for confounding factors.

Earlier observational studies suggested that maternal hyperglycemia may affect fetal brain development through mechanisms such as oxidative stress or inflammatory pathways. In the present cohort, however, the corrected BSID-CR DQ score values and revised DQ category distribution continue to show that GDM was not independently associated with early developmental outcomes. These corrections affect the absolute BSID-CR DQ score values, DQ category distribution, Figure 2, Table 3, and the linear regression model, but do not alter the study design, analytic framework, or main conclusion.

Importantly, our cohort included detailed characterization of GDM severity, metabolic control, and treatment modality. Even after stratification by pharmacologic treatment requirement and glycemic control status, no significant differences in neurodevelopmental outcomes were observed. These findings suggest that, within this clinically monitored population, gestational diabetes across varying levels of severity, was not independently associated with early cognitive impairment.

Interestingly, our study revealed absence of significant associations between cognitive and developmental function and a range of maternal and neonatal variables including maternal age, BMI, mode of delivery and parity indicating the complex and multifactorial nature of cognitive development. Logistic regression analysis showed that gender had no significant association with cognitive function and neurodevelopment of infants less than 2 years which aligns with findings by Ozkan et al. in study of infant between 3 and 30 months (31). Additionally, Mkwambe et al., showed similar findings regarding gender in infants at their first year (20). In contrast, Xiang et al., found that male infants born to mother with diabetes are at higher risk for developmental and behavioral impairment compared to female infants (12). Regarding mode of delivery there was no obvious association with cognitive impairment. These findings were consistent with previous studies which reported similar findings (32, 33). Furthermore, parity and obstetric history were not associated with cognitive impairment in infancy which contrasts the evidence reported by Mkwambe et al., demonstrating that primiparous mothers showed higher neurodevelopmental defects compared to multiparous women (20). However, Abubakar et al., proposed that each successive pregnancies increase the probability of adverse developmental outcomes in younger children (34).

### Strengthens and clinical implications

This study highlighted the association between GDM and neurodevelopmental outcomes in infancy and exhibited several notable strengths, including a well-balanced cohort with a comprehensive adjustment for a broad range of maternal and neonatal variables. Importantly, cognitive and psychiatric outcomes were evaluated using validated tools, enhancing the clinical relevance of the results. Despite expectations of potential developmental risks associated with GDM, the study revealed no significant differences in cognitive disorders or scores between children of mothers with and without GDM. This finding holds critical clinical implications, suggesting that GDM, when managed effectively, may not independently predispose offspring to cognitive impairments. Thus, the study supports current clinical strategies that emphasize tight glycemic control during pregnancy, while also reassuring healthcare providers and parents regarding long-term neurodevelopmental outcomes in GDM-exposed offspring.

### Limitations

Despite these strengthens, our study showed some limitations to acknowledge. Firstly, the retrospective cohort design relies on existing records and perinatal reports, which may be subject to recall bias or incomplete documentation. The relatively small sample size could have limited statistical power, affecting the detection of subtle differences. Although detailed glycemic measures and treatment modalities were available and incorporated into subgroup analyses, residual confounding related to duration of hyperglycemia or unmeasured metabolic variability cannot be entirely excluded. Additionally, cognitive assessments conducted within the first 2 years of life may not fully predict long-term developmental trajectories. Variability in metabolic control may influence neurodevelopmental outcomes and could not be examined in this analysis. Neurodevelopmental and psychiatric evaluations conducted within the first 2 years of life primarily reflect early developmental performance and may not reliably capture disorders that manifest later in childhood. Future prospective studies and clinical trials are required to get conclusive and supportive evidence about the impact of GDM and cognitive and developmental outcomes in childhood and to overcome the discrepancy of findings of current literature.

## Conclusion

In conclusion, within the limitations of this retrospective analysis, no significant differences were observed in early neurodevelopmental outcomes between infants born to mothers with and without GDM. Adjusted analyses confirmed that GDM is not considered as independent predictor of cognitive and developmental outcomes in infancy. Continuous monitoring and early developmental screening remain important for all infants especially with diseased mothers.

## Data Availability

The original contributions presented in the study are included in the article/Supplementary Material, further inquiries can be directed to the corresponding author.

## References

[1] Stogianni A Lendahls L Landin-Olsson M Thunander M. Obstetric and perinatal outcomes in pregnancies complicated by diabetes, and control pregnancies, in Kronoberg, Sweden. BMC Pregnancy Childbirth. (2019) 19(1):159. 10.1186/s12884-019-2269-8 31064335 PMC6505274

[2] Saeedi P Petersohn I Salpea P Malanda B Karuranga S Unwin N. Global and regional diabetes prevalence estimates for 2019 and projections for 2030 and 2045: results from the International Diabetes Federation Diabetes Atlas, 9th edition. Diabetes Res Clin Pract. (2019) 157:107843. 10.1016/j.diabres.2019.107843 31518657

[3] The International Diabetes Federation. Prevalence of gestational diabetes mellitus (GDM), % [Internet]. Diabetes Atlas. Available online at: https://diabetesatlas.org/data-by-indicator/hyperglycaemia-in-pregnancy-hip-20-49-y/prevalence-of-gestational-diabetes-mellitus-gdm/ (Accessed July 29, 2025).

[4] Qian L Xin T Xu W Zhu J. Effect of maternal gestational diabetes mellitus on neurodevelopment in late preterm infants at the corrected age of 12 months. Sci Rep. (2025) 15(1):14139. 10.1038/s41598-025-98987-w 40269066 PMC12019377

[5] The HAPO Study Cooperative Research Group. Hyperglycemia and adverse pregnancy outcome (HAPO) study. Diabetes. (2009) 58(2):453–9. 10.2337/db08-1112 19011170 PMC2628620

[6] Mattila I Nolvi S Kataja EL Tuulari JJ Korja R Scheinin NM. Gestational diabetes mellitus and children’s social-emotional development, behavioral problems, and psychological adjustment. Pediatr Res. (2025) 99:126–33. 10.1038/s41390-025-04191-x 40494867 PMC12920097

[7] Johns EC Denison FC Norman JE Reynolds RM. Gestational diabetes mellitus: mechanisms, treatment, and complications. Trends Endocrinol Metab. (2018) 29(11):743–54. 10.1016/j.tem.2018.09.004 30297319

[8] Ali DS Davern R Rutter E Coveney C Devine H Walsh JM. Pre-gestational diabetes and pregnancy outcomes. Diabetes Ther. (2020) 11(12):2873–85. 10.1007/s13300-020-00932-9 33010001 PMC7644712

[9] Shah R Dai DWT Alsweiler JM Brown GTL Chase JG Gamble GD. Association of neonatal hypoglycemia with academic performance in mid-childhood. J Am Med Assoc. (2022) 327(12):1158. 10.1001/jama.2022.0992 PMC8941348 35315886

[10] Akinseye A Pylypjuk C Moddemann D Afifi J Banihani R Aziz K. Maternal diabetes and neurodevelopmental outcomes of infants born before 29 weeks’ gestation. J Pediatr. (2025) 276:114319. 10.1016/j.jpeds.2024.114319 39306321

[11] Pretorius RA Avraam D Guxens M Julvez J Harris JR Nader JT. Is maternal diabetes during pregnancy associated with neurodevelopmental, cognitive and behavioural outcomes in children? Insights from individual participant data meta-analysis in ten birth cohorts. BMC Pediatr. (2025) 25(1):76. 10.1186/s12887-024-05365-y 39885386 PMC11783732

[12] Xiang AH Wang X Martinez MP Page K Buchanan TA Feldman RK. Maternal type 1 diabetes and risk of autism in offspring. J Am Med Assoc. (2018) 320(1):89. 10.1001/jama.2018.7614 PMC6134431 29936530

[13] Arabiat D Al Jabery M Jenkins M Kemp V Whitehead L Adams G. Language abilities in children born to mothers diagnosed with diabetes: a systematic review and meta-analysis. Early Hum Dev. (2021) 159:105420. 10.1016/j.earlhumdev.2021.105420 34247025

[14] Lappas M Hiden U Desoye G Froehlich J de Mouzon SH Jawerbaum A. The role of oxidative stress in the pathophysiology of gestational diabetes mellitus. Antioxid Redox Signal. (2011) 15(12):3061–100. 10.1089/ars.2010.3765 21675877

[15] Calvo MJ Parra H Santeliz R Bautista J Luzardo E Villasmil N. The placental role in gestational diabetes mellitus: a molecular perspective. touchREV Endocrinol. (2024) 20(1):10–8. 10.17925/EE.2024.20.1.5 38812661 PMC11132656

[16] Márquez-Valadez B Valle-Bautista R García-López G Díaz NF Molina-Hernández A. Maternal diabetes and fetal programming toward neurological diseases: beyond neural tube defects. Front Endocrinol. (2018) 9:664. 10.3389/fendo.2018.00664 PMC6243582 30483218

[17] Gluckman PD Hanson MA Low FM. The role of developmental plasticity and epigenetics in human health. Birth Defects Res C Embryo Today. (2011) 93(1):12–8. 10.1002/bdrc.20198 21425438

[18] Chu AHY Godfrey KM. Gestational diabetes mellitus and developmental programming. Ann Nutr Metab. (2020) 76(Suppl. 3):4–15. 10.1159/000509902 33465774 PMC7116810

[19] Al Bekai E Beaini CE Kalout K Safieddine O Semaan S Sahyoun F. The hidden impact of gestational diabetes: unveiling offspring complications and long-term effects. Life. (2025) 15(3):440. 10.3390/life15030440 40141785 PMC11944258

[20] Mkwambe MC Deng Y Zhao D. The impact of gestational diabetes mellitus on neurodevelopmental outcomes in infants. Open J Pediatr. (2025) 15(02):150–76. 10.4236/ojped.2025.152015

[21] Xu T Faleschini S Rifas-Shiman SL Monthé-Drèze C Oken E Hivert M. Maternal glucose tolerance in pregnancy and child cognitive and behavioural problems in early and mid-childhood. Paediatr Perinat Epidemiol. (2021) 35(1):109–19. 10.1111/ppe.12710 32885485 PMC7877074

[22] Balasundaram P Avulakunta ID. Bayley scales of infant and toddler development. In: Avulakunta ID, editor. StatPearls. Treasure Island (FL): StatPearls Publishing (2025). 33620792

[23] IBM Corp. IBM SPSS. Armonk, NY: IBM Corp (2020) (IBM SPSS Statistics for Windows).

[24] Ornoy A Ratzon N Greenbaum C Wolf A Dulitzky M. School-age children born to diabetic mothers and to mothers with gestational diabetes exhibit a high rate of inattention and fine and gross motor impairment. J Pediatr Endocrinol Metab. (2001) 14(Supplement):681–9. 10.1515/JPEM.2001.14.S1.681 11393563

[25] Marks DJ. Exposure to gestational diabetes mellitus and low socioeconomic status: effects on neurocognitive development and risk of attention-deficit/hyperactivity disorder in offspring. Arch Pediatr Adolesc Med. (2012) 166(4):337. 10.1001/archpediatrics.2011.784 22213602 PMC5959273

[26] Faleschini S Doyon M Arguin M Lepage JF Tiemeier H Van Lieshout RJ. Maternal hyperglycemia in pregnancy and offspring internalizing and externalizing behaviors. Matern Child Health J. (2023) 27(10):1765–73. 10.1007/s10995-023-03706-4 37296332

[27] Hochstedler KA Bell G Park H Ghassabian A Bell EM Sundaram R. Gestational age at birth and risk of developmental delay: the upstate KIDS study. Am J Perinatol. (2021) 38(10):1088–95. 10.1055/s-0040-1702937 32143225 PMC7507972

[28] Camerota M Lester BM McGowan EC Carter BS Check J Dansereau LM. Contributions of prenatal risk factors and neonatal epigenetics to cognitive outcome in children born very preterm. Dev Psychol. (2024) 60(9):1606–19. 10.1037/dev0001709 38358663 PMC11618652

[29] Rowland J Wilson CA. The association between gestational diabetes and ASD and ADHD: a systematic review and meta-analysis. Sci Rep. (2021) 11(1):5136. 10.1038/s41598-021-84573-3 33664319 PMC7933135

[30] Monteiro LJ Norman JE Rice GE Illanes SE. Fetal programming and gestational diabetes mellitus. Placenta. (2016) 48:S54–60. 10.1016/j.placenta.2015.11.015 26724985

[31] Ozkan M Senel S Arslan EA Karacan CD. The socioeconomic and biological risk factors for developmental delay in early childhood. Eur J Pediatr. (2012) 171(12):1815–21. 10.1007/s00431-012-1826-1 22983025

[32] Huang Y Chang J Chen C Peng C Hsu C Ko MH. Association of mode of delivery with short-term and neurodevelopmental outcomes in periviable singleton infants: a nationwide database study. Int J Gynecol Obstet. (2023) 163(1):307–14. 10.1002/ijgo.14833 37170688

[33] Wolf HT Weber T Schmidt S Norman M Varendi H Piedvache A. Mode of delivery and adverse short- and long-term outcomes in vertex-presenting very preterm born infants: a European population-based prospective cohort study. J Perinat Med. (2021) 49(7):923–31. 10.1515/jpm-2020-0468 34280959

[34] Abubakar A Holding P Van De Vijver FJR Newton C Van Baar A. Children at risk for developmental delay can be recognised by stunting, being underweight, ill health, little maternal schooling or high gravidity. J Child Psychol Psychiatry. (2010) 51(6):652–9. 10.1111/j.1469-7610.2009.02193.x 19951363 PMC2919164

