# Supplementary material for: Gestational diabetes and its influence on early neurodevelopment in infants: a retrospective analysis
Source: Front Pediatr. 2026 Aug 19;14:1756844. doi: 10.3389/fped.2026.1756844 (PMC13534006; doi:10.3389/fped.2026.1756844)
Supplement: Supplementary file 1 [file Table1.docx]

Table S1. Gestational Diabetes Severity and Glycemic Control Characteristics (n = 145)

| Variable | Value |
| --- | --- |
| OGTT Fasting Glucose (mg/dL), median (Q1–Q3) | 96 (91–102) |
| OGTT 1-hour Glucose (mg/dL), median (Q1–Q3) | 182 (168–196) |
| OGTT 2-hour Glucose (mg/dL), median (Q1–Q3) | 154 (142–168) |
| HbA1c During Pregnancy (%), median (Q1–Q3) | 5.8 (5.5–6.2) |
|  |  |
| GDM Severity Classification, n (%) |  |
| Mild (single abnormal OGTT value) | 72 (49.7%) |
| Moderate (≥2 abnormal OGTT values) | 48 (33.1%) |
| Severe (requiring pharmacologic therapy) | 25 (17.2%) |
|  |  |
| Treatment Modality, n (%) |  |
| Diet-controlled | 108 (74.5%) |
| Pharmacologic therapy (insulin/oral agents) | 37 (25.5%) |
|  |  |
| Glycemic Control Status During Pregnancy, n (%) |  |
| Adequate control | 118 (81.4%) |
| Suboptimal control | 27 (18.6%) |

Table S2. Subgroup Analysis of Neurodevelopmental Outcomes by GDM Treatment Modality

| Outcome | Diet-Controlled GDM (n = 108) | Pharmacologic Therapy (n = 37) | P-value |
| --- | --- | --- | --- |
| Any Cognitive/Psychiatric Disorder, n (%) | 35 (32.4%) | 13 (35.1%) | 0.74 |
| Autism Spectrum Disorder, n (%) | 1 (0.9%) | 1 (2.7%) | 0.46 |
| ADHD, n (%) | 9 (8.3%) | 4 (10.8%) | 0.63 |
| Mild Cognitive Delay, n (%) | 9 (8.3%) | 3 (8.1%) | 0.97 |
| Speech Delay, n (%) | 16 (14.8%) | 5 (13.5%) | 0.84 |
|  |  |  |  |
| Bayley-4 Score, median (Q1–Q3) | 74.6 (64.2–86.1) | 72.8 (62.9–84.5) | 0.58 |
